# Statin use and endometrial cancer risk: a meta-analysis

**DOI:** 10.18632/oncotarget.18658

**Published:** 2017-06-27

**Authors:** Jing Yang, Qiaoling Zhu, Qiao Liu, Yingxia Wang, Weimin Xie, Lili Hu

**Affiliations:** ^1^ Department of Obstetrics and Gynecology, Peking Union Medical College Hospital, Chinese Academy of Medical Sciences and Peking Union Medical College, Beijing, China; ^2^ Department of Obstetrics and Gynecology, Maternal and Child Health Care Hospital of Hunan Province, Changsha, China; ^3^ Department of Obstetrics and Gynecology, Jilin Central Hospital, Jilin, China

**Keywords:** statin, endometrial cancer, cancer risk, meta-analysis

## Abstract

Several studies have evaluated the association between statin use and endometrial cancer risk. We carried out a meta-analysis of randomized controlled trials (RCTs) and non-randomized studies to evaluate the effect of statins on endometrial cancer risk. A comprehensive search of electronic databases, conference abstracts and clinical trial registers was conducted for published and unpublished results. Studies that evaluated exposure to statins and endometrial cancer risk were considered. Pooled relative risks (RRs) with 95% confidence intervals (CIs) were calculated using either a fixed-effects or a random-effects model. Two RCTs and eleven non-randomized studies (four cohort and seven case-control studies) involving 9,517 cases of endometrial cancer were included in the analysis. There was no evidence of an association between statin use and endometrial cancer risk either among RCTs (RR, 0.72; 95% CI, 0.19 to 2.67) or among non-randomized studies (RR, 0.94; 95% CI, 0.82 to 1.07). Combined analysis of all included studies also showed that statin use did not significantly affect endometrial cancer risk (RR, 0.94; 95% CI, 0.82 to 1.07). The sensitivity analysis confirmed the stability of our results. Our findings do not support a protective effect of statins against endometrial cancer at the population level.

## INTRODUCTION

Endometrial cancer is the most common malignancy of the female genital tract in the United States, with 60,050 new cases estimated in 2016 [1]. Obesity is one of the well known risk factors for endometrial cancer, whereas the relation between several obesity-related comorbid conditions such as hypertension and dyslipidemia and endometrial cancer remains unclear. Given the poor outcome of advanced-stage endometrial cancer, it is imperative to assess the factors that can help to decrease endometial cancer risk.

Statins or 3-Hydroxy-3-methylglutaryl-coenzyme A (HMG-CoA) reductase inhibitors are widely used to reduce plasma cholesterol levels and prevent cardiovascular diseases. Statins block the HMG-CoA reductase, the enzyme required for conversion of HMG-CoA to the cholesterol precursor mevalonic acid [2]. Satins are thought to lead a critical changes in cellular functions through inhibition of the mevalonic acid pathway.

Debate regarding the association between statin use and cancer risk is ongoing. In a review of rodent carcinogenicity tests, an association betweeen lipid-lowering drugs, including statins, and increased cancer incidence was reported in rats and mice [3]. In contrast, statins have been shown to suppress cell proliferation [4], induce apoptosis [5], inhibit angiogenesis [6], and inhibit metastatic mechanism [7] *in vitro* and *in vivo* in many recent laboratory studies, which prevent cancer growth and development. Statins have anti-proliferative and anti-metastatic effects in endometrial cancer cells, possibly through regulation of the MAPK and AKT/mTOR pathways [8, 9]. Even though a climbing amount of laboratory studies have shown the anticancer effects of statins in various cell lines, the results regarding the chemopreventive role of statins against cancers in observational studies and clinical trials are inconsistent. Several meta-analyses have suggested that the use of statins is associated with a lower risk of site-specific cancers, including non-Hodgkin lymphoma, esophageal, liver and gastric cancer [10–13], while others have not shown this effect, including breast, colorectal, pancreatic, skin, and prostate cancer [14–18].

Recently, a number of studies have also evaluated the relationship between statin use and endometrial cancer risk [19–31]. However, the existing findings from these studies are controversial, with no beneficial effect in the majority of studies [20–24, 26–28, 31], whereas others supported a reduced risk [19, 25, 29, 30]. To better understand the effect of statins on endometrial cancer risk, we carried out a meta-analysis of available randomized controlled trials (RCTs) and non-randomized studies on the subject.

## RESULTS

### Study selection

**Figure 1:**
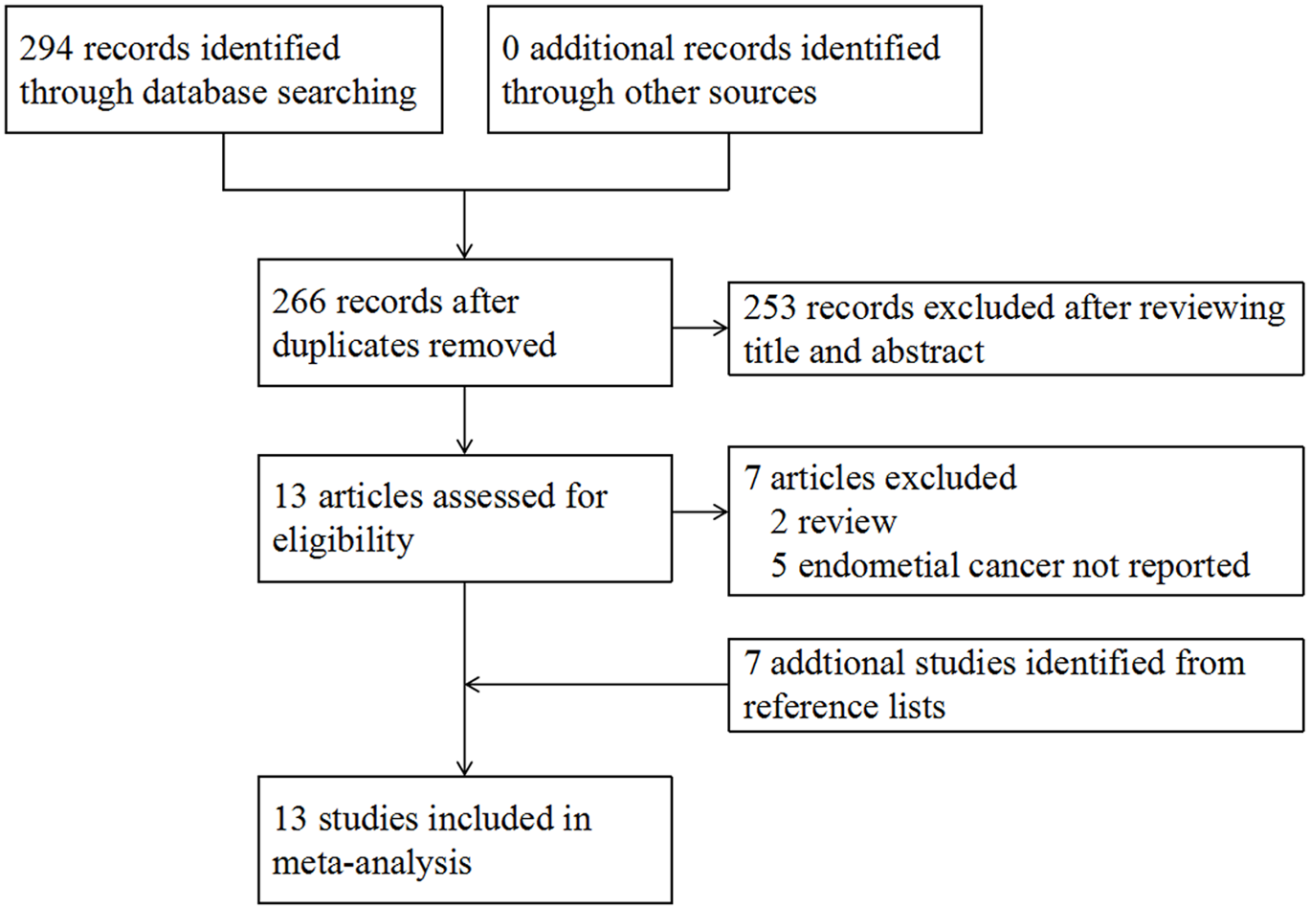
Study flow diagram

In our initial search, 294 records were identified through database searching, no additional records were found from conference proceedings or trial registers. After screening the titles and abstracts, 13 potentially relevant papers were retrieved for full-text review. Of these, we excluded 7 studies for the following reasons: 2 were narrative review studies [32, 33], 5 did not reported endometrial cancer risk [34–38]. Seven studies were retrieved from reference lists [19–25]. Finally, a total of 13 studies that met our eligibility criteria were included in the meta-analysis [19–31], with 2 RCTs [20, 22], 4 cohort studies [23, 24, 27, 29], and 7 case-control studies [19, 21, 25, 26, 28, 30, 31]. The flow diagram for study selection is shown in Figure 1.

The studies were published between 2001 and 2016. Of these, 7 studies were carried out in North America [19, 20, 23, 26–29], 4 in Europe [21, 22, 24, 31], and 2 in Asia [25, 30]. A total of 9,517 cases of endometrial cancer were involved. The two RCTs were double-blind, plocebo-controlled trials of monotherapy with a statin [20, 22]. In all non-randomized studies, the estimates of effect (RR) were adjusted for potential confounding factors [19, 21, 23–31]. The characteristics of the included RCTs and non-randomized studies are shown in Tables 1 and 2, respectively.

**Table 1:** Randomized controlled trials Included in the meta-analysis

Study	Year	Study location	Agent	Follow-up (years)	All female patients	Incident endometrial cancer		RR	95% CI
						Statin group	Control group		
Clearfield et al. [20]	2001	United States	Lovastatin	Mean: 5.2	997	1 of 499	3 of 498	0.33	0.03 to 3.19
Strandberg et al. [22]	2004	Nordic countries	Simvastatin	Median: 10.4	827	3 of 407	3 of 420	1.03	0.21 to 5.08

RR, relative risk; CI, confidence interval.

**Table 2:** Non-randomized studies included in the meta-analysis

Study	Study location	Study design	All female patients	EC patients	RR	95% CI	Control for potential confounding factors^b^	Study quality (NOS value)
Blais et al., 2000 [19]	Canada	C-C	286	26	0.3	0.11 to 0.81	1-6	6
Kaye et al., 2004 [21]	United Kingdom	C-C	8,978	24	0.5	0.1 to 0.9	1, 7, 8	8
Friedman et al., 2008 [23]	United States	Cohort	NR	199	1.13	0.98 to 1.31	3	6
Haukka et al., 2010 [24]	Finland	Cohort	473,302	1,721	1.05	0.95 to 1.15	1, 9	7
Leung et al., 2013 [25]	China	C-C	18,055	222	0.43	0.196 to 0.933	1,5, 10-12	7
Coogan et al., 2007 [26]	United States	C-C	4,641	220	1.3	0.7 to 2.4	1,7,8,11,13-17	5
Yu et al., 2009 [27]	United States	Cohort	73,336	568	0.67	0.39 to 1.17	1,5,7,18,19	8
Fortuny et al., 2009 [28]	United States	C-C	936	469	1.3	0.8 to 2.1	1,7,8,10,13, 14,20-24	7
Jacobs et al., 2011 [29]^a^	United States	Cohort	73,196	461	1.17 1.11 0.65	0.76 to 1.81 0.84 to 1.42 0.45 to 0.94	1,7,8,10,11, 13,14, 18,25-28	8
Lavie et al., 2013 [30]	Israel	C-C	430	215	0.55	0.37 to 0.81	7,10,14,24,25,29-31	8
Sperling et al., 2016 [31]	Denmark	C-C	77,509	5,382	1.03	0.94 to 1.14	1,7,10,13,18,23,32	9

EC, endometrial cancer; RR, relative risk; CI, confidence interval; NOS, Newcastle–Ottawa scale; C-C, case control; NR, not reported.

^a^ The RRs and 95% CIs were presented in patients with former use of statin, current short-term (< 5 years) use and long-term (≥ 5 years) use compared with never users, respectively.

^b^ Confounding factors: 1, age; 2, previous neoplasm; 3, year of entry; 4, use of fibric acids; 5, use of other lipid-reducing agents; 6, the comorbidity score; 7, BMI; 8, smoking; 9, follow-up period; 10, use of hormone therapy; 11, use of nonsteroidal anti-inflammatory drug; 12, comorbidities at cancer diagnosis; 13, education; 14, race; 15, use of alcohol; 16, interview year; 17, study center; 18, diabetes; 19, high-triglyceride drug use; 20, menarche; 21, use of oral contraceptives; 22, age at menopause; 23, parity; 24, family history of endometrial cancer; 25, physical activity; 26, history of elevated cholesterol; 27, heart disease; 28, hypertension; 29, fruit and vegetable consumption; 30, duration of breast feeding; 31, age at 1st pregnancy; 32, chronic obstructive pulmonary disease.

### Quality assessment results

**Figure 2:**
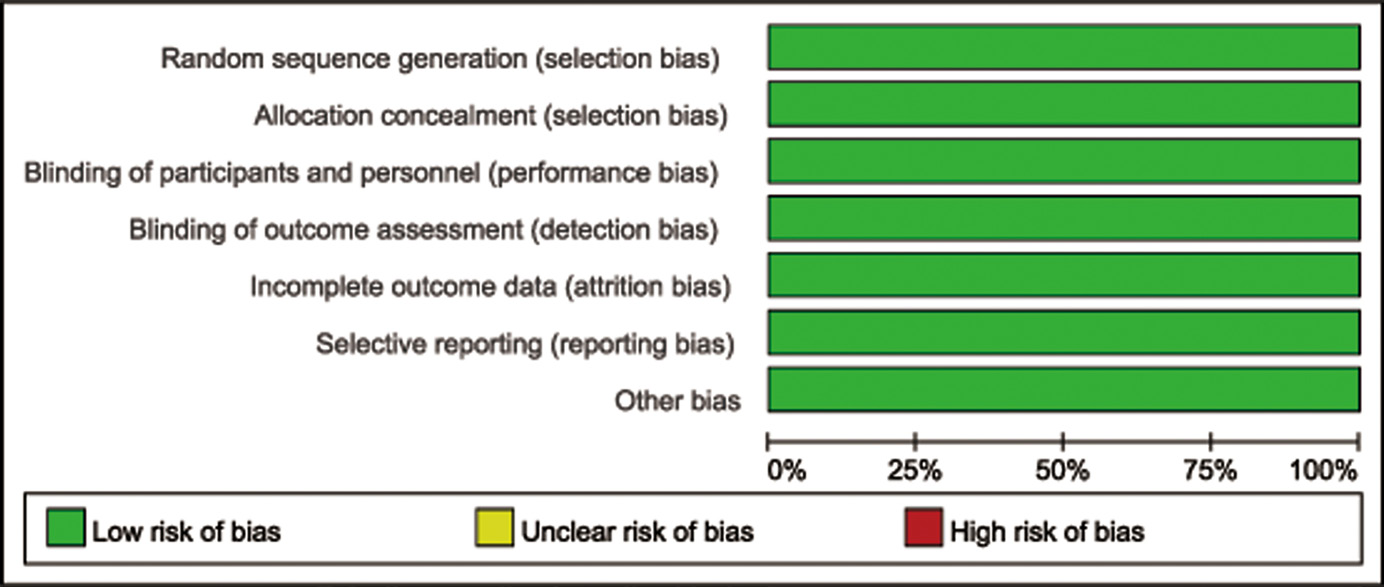
Risk of bias graph: review authorsʼ judgements about each risk of bias item presented as percentages across all included studies

The risk of bias assessment for the included 2 RCTs is presented in Figure 2. Both of the two studies were considered at low risk of selection bias, performance bias, detection bias, attrition bias, and reporting bias. With regard to the quality of non-randomized studies, the NOS values ranged from 5 to 9 stars: 1study was awarded 5 stars, 2 studies were awarded 6 stars, 3 studies were awarded 7 stars, 4 studies were awarded 8 stars, and 1study was awarded 9 stars (Table 2).

### Meta-analysis of RCTs

**Figure 3:**
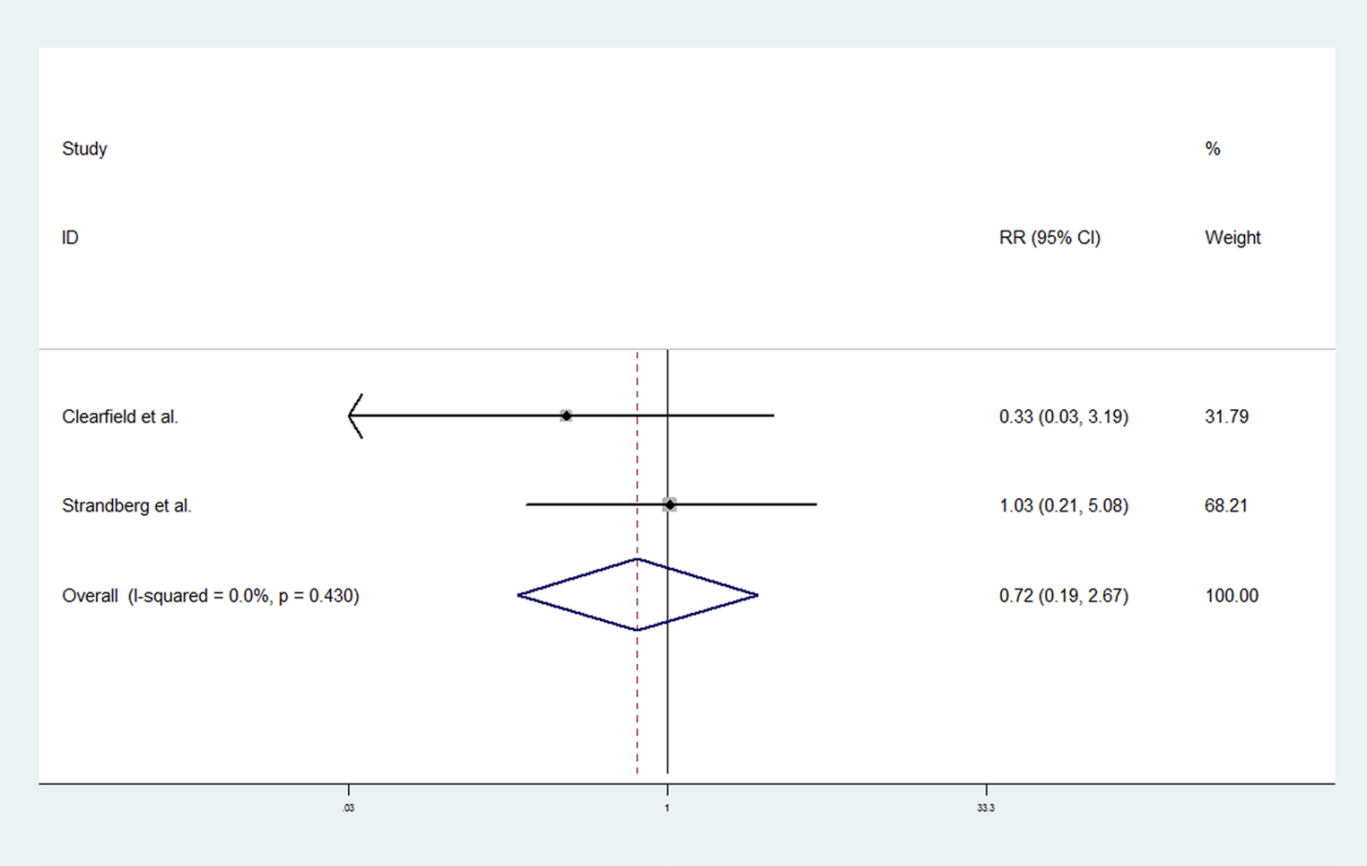
Forest plot of statin use and endometrial cancer risk from RCTs Pooled effect estimate is from a fixed-effects model.

Two RCTs of statins contributed to the analysis [20, 22]. A total of 1,824 women participated in these trials: 906 in statin group and 918 in control group. Median follow-up during the trial were 5.2 years [20] and 10.4 years [22], respectively. The pooled data showed that the association of statin use with endometrial cancer risk was not statistically significant (RR, 0.72; 95% CI, 0.19–2.67). The Chi-square test resulted in a *p* value of 0.43, and the corresponding *I*^*2*^ was 0.0%, both indicating that there was no evidence of heterogeneity (Figure 3).

### Meta-analysis of non-randomized studies

**Figure 4:**
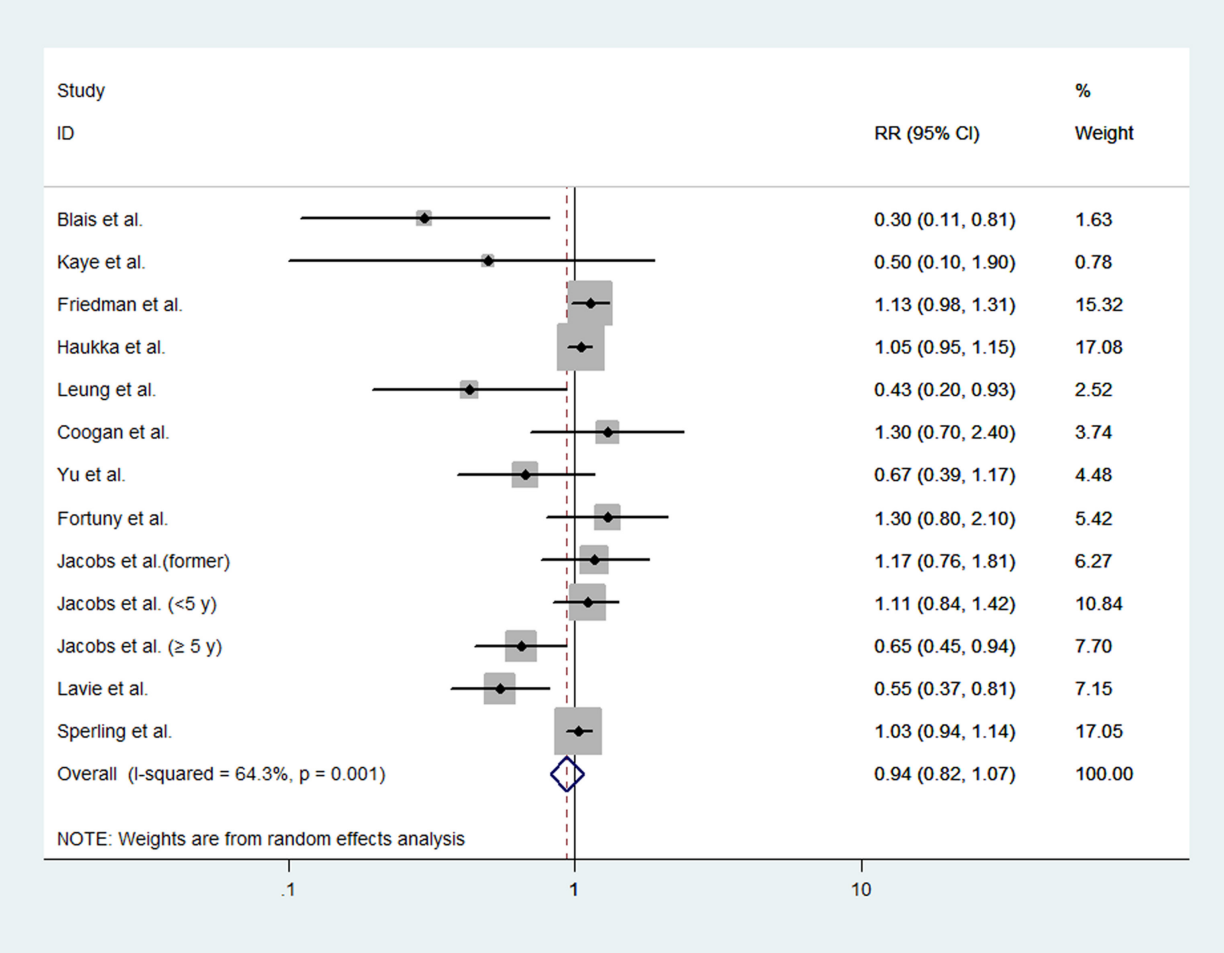
Forest plot of statin use and endometrial cancer risk from non-randomized studies Pooled effect estimate is from a random-effects model.

Four cohort studies [23, 24, 27, 29] and 7 case-control studies [19, 21, 25, 26, 28, 30, 31] evaluated exposure to statins and endometrial cancer risk. The pooled analysis included a total of 9,507 cases of endometrial cancer in these eleven studies. The pooled data showed that statin use did not significantly affect the risk of endometrial cancer (RR, 0.94; 95% CI, 0.82 to 1.07). The Chi-square test resulted in a *p* value of 0.001, and the corresponding *I*^*2*^ was 64.3%, both indicating that the heterogeneity among the studies was considerable (Figure 4).

**Figure 5:**
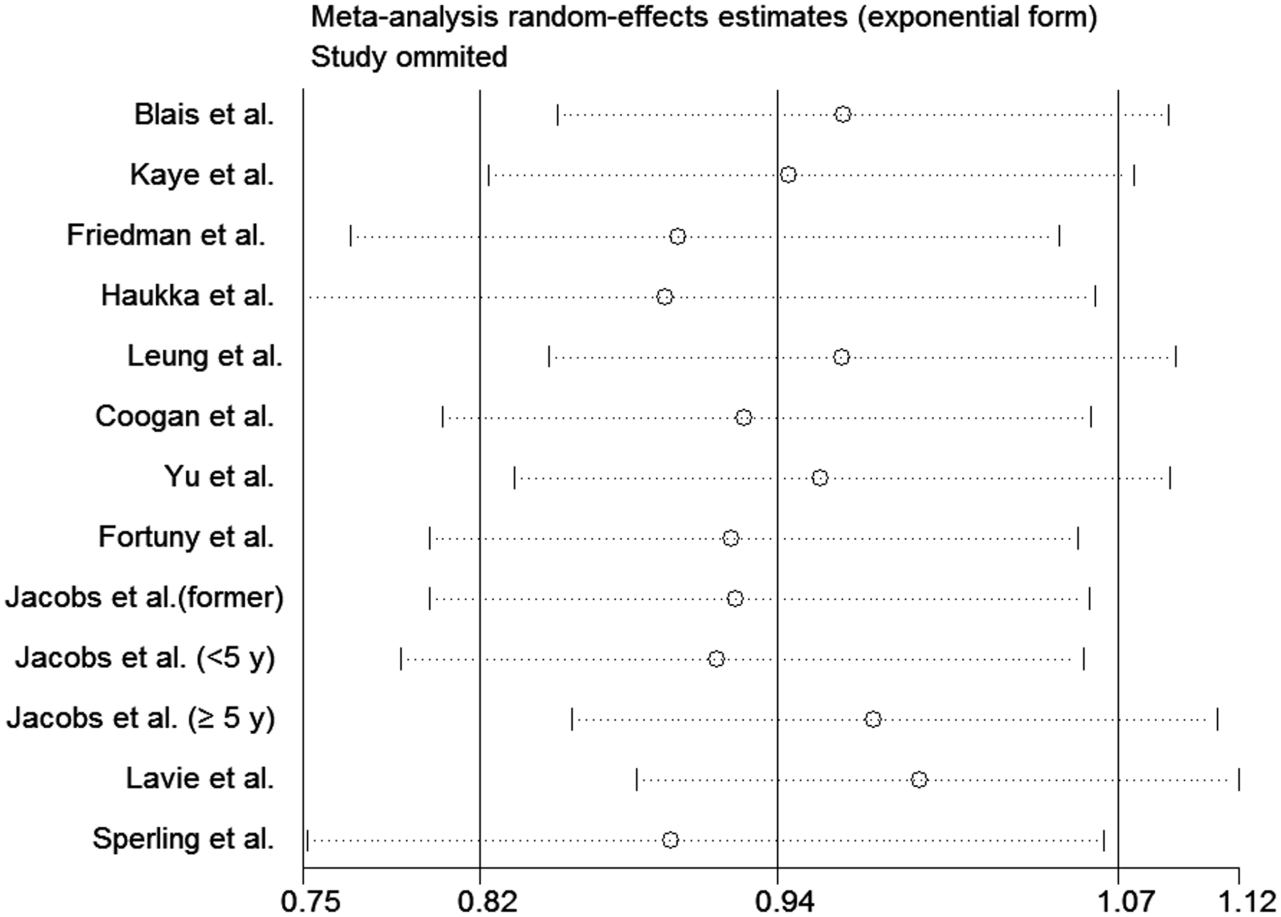
Sensitivity analysis of statin use and endometrial cancer risk from non-randomized studies

We performed stratifying analyses of studies on the basis of study design and study location (Table 3). We found no association between statin use and endometrial cancer risk among cohort studies (RR, 1.01; 95% CI, 0.88 to 1.16) or case-control studies (RR, 0.77; 95% CI, 0.54 to 1.11). On stratified analysis based on study location, statin use was associated with a significant reduction in endometrial cancer risk among 2 studies carried out in Asia (RR, 0.52; 95% CI, 0.37 to 0.74). However, the association of the use of statins with endometial cancer risk was not statistically significant among 6 studies carried out in North America (RR, 0.97; 95% CI, 0.78 to 1.20) and 4 in Europe (RR, 1.04; 95% CI, 0.97 to 1.11). In sensitivity analysis, each study was excluded and its influence was evaluated by repeating the primary analysis. The analysis confirmed the stability of our results because that none of the individual studies markedly affected the pooled effect (Figure 5).

**Table 3:** Subgroup analysis of non-randomized studies

Subgroups	No. of studies	No. of cases	RR (95% CI)	Heterogeneity		Heterogeneity between subgroups, *p* value
				*I*^*2*^ (%)	*p* value	
Study design						
Cohort studies	4	6558	1.01 (0.88 to 1.16)	52.3	0.063	
Case-control studies	7	2949	0.77 (0.54 to 1.11)	72.7	0.001	0.289
Study location						
North America	6	1943	0.97 (0.78 to 1.20)	60.5	0.013	
Europe	3	7127	1.04 (0.97 to 1.11)	0.0	0.599	
Asia	2	437	0.52 (0.37 to 0.74)	0.0	0.581	0.001

### Overall analysis

**Figure 6:**
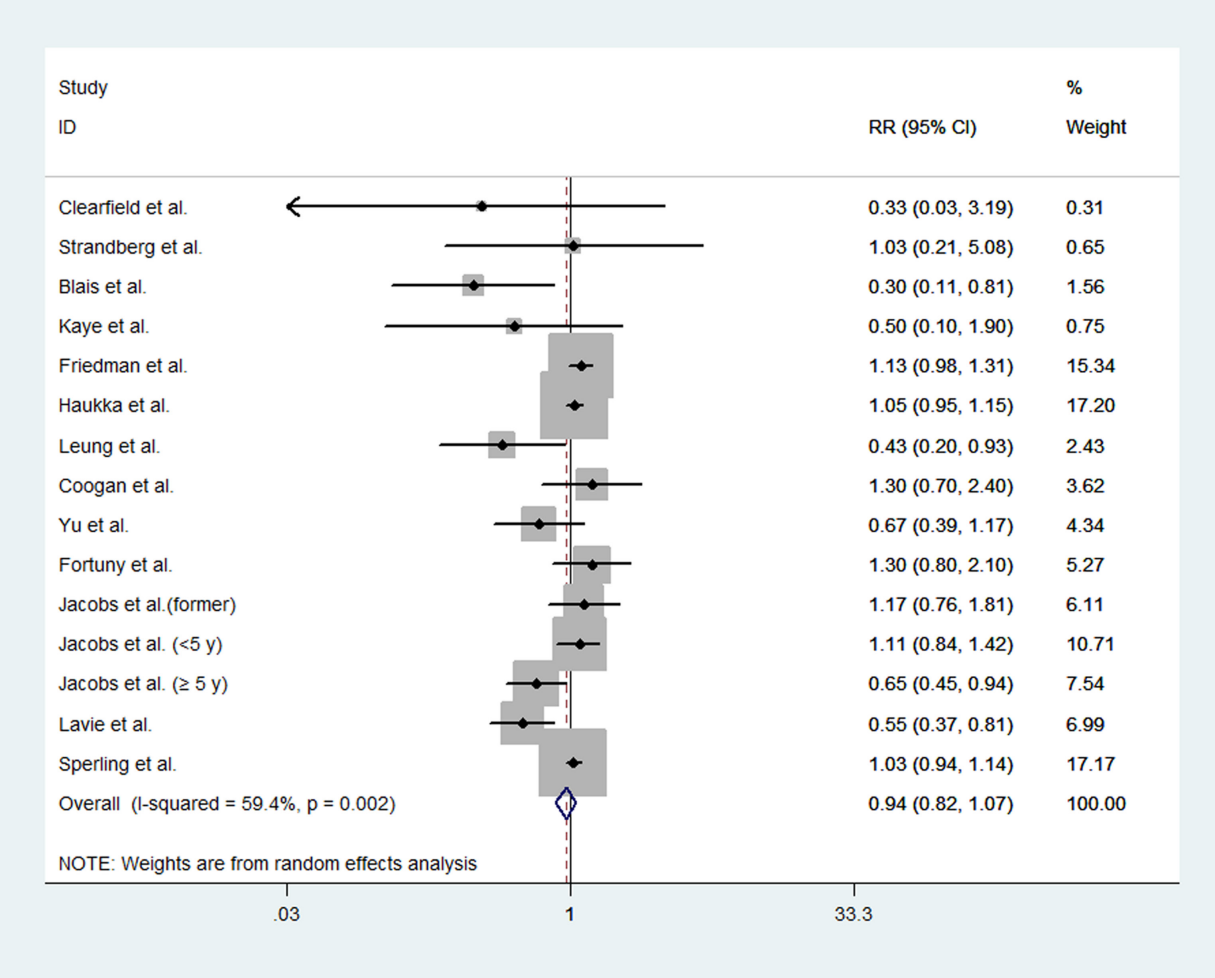
Forest plot of statin use and endometrial cancer risk from all included studies Pooled effect estimate is from a random-effects model.

We performed a combined analysis of RCTs and non-randomized studies. The pooled data showed that statin use did not significantly affect endometrial cancer risk (RR, 0.94; 95% CI, 0.82 to 1.07). The Chi-square test resulted in a *p* value of 0.002, and the corresponding *I*^*2*^ was 59.4%, indicating moderate heterogeneity across the studies (Figure 6).

## DISCUSSION

To date, most of the available results of statin use and endometrial cancer risk are from observational studies, only two RCTs reported endometrial cancer risk as a secondary end point [20, 22]. Thus, we carried out this meta-analysis by pooling the results from both available RCTs and non-randomized studies to evaluate specifically the association between statin use and endometrial cancer risk.

In the present meta-analysis, both the synthesis of RCTs and non-randomized studies showed no evidence that statin use is associated with a significant relative decreased or increased risk of endometrial cancer. The results were not affected in subgroup analysis regarding study design and sensitivity analysis, which confirmed the validity of the conclusion. In addition, several meta-analyses also have found that statin use does not substantially affect overall cancer risk [39–41]. Although it was observed that statin use is associated a decreased risk of endometrial cancer in Asian population, no conclusion should be made because there was only two case-control studies carried out in this region [25, 30]. Lung et al [25] noted that the cases of statin use had a higher frequency of use of cardiovascular drugs in their study, such as aspirin. Two recent meta-analyses [42, 43] have suggested that aspirin use was associated with a significant reduced risk of endometrial cancer, which indicate that aspirin use may be one of confounding factors. However, the adjusted RR and 95% CI provided by Lung et al did not include aspirin use as a confounding factor [25], which may have influenced the results.

The present study has several merits. We performed a comprehensive search, including electronic databases, conference abstracts and clinical trial registers for published and unpublished results. We sought articles without language restrictions and did not exclude any article regarding methodological characteristics or place of publication. Hence, the likelihood of important selection or publication bias was small in the review process. Systematic reviews have found that the estimates of effect derived from RCTs and non-randomized studies are likely to be different [44]. Failure to use adequately concealed random allocation can cause the outcomes of interest to seem either larger or smaller than they really are. We included both RCTs and non-randomized studies and presented the pooled data of them and a combined analysis of all included studies. It was noteworthy that the findings were similar in both meta-analyses of RCTs and non-randomized studies, which strengthened our confidence in the validity of the conclusion.

Nevertheless, the present meta-analysis had several limitations. First, the available relevant RCTs were limited, which mainly investigated cardiovascular outcomes and assessed the incidence of endometrial cancer only as a secondary end point. Second, there was significant heterogeneity in meta-analysis of non-randomized studies, which might partly due to the extra sources of methodological diversity and bias compared to RCTs. Third, all studies did not adjust for the same potential confounding factors. In addition, the limited data made it impractical to evaluate dose, duration, and type effects of statins.

In conclusion, despite the chemopreventive potential of statins demonstrated in several laboratory studies, our findings do not support a protective effect of statins against endometrial cancer at the population level. Due to the limited data regarding the association between statin use and endometrial cancer risk, further high quality clinical studies are needed to validate our findings.

## MATERIALS AND METHODS

This meta-analysis was prepared according to the Preferred Reporting Items for Systematic Reviews and Meta-Analyses (PRISMA) statement [45].

### Search strategy

We searched for all published and unpublished randomized controlled trials (RCTs) and non-randomized studies, without language restrictions. We searched the following electronic databases from inception to 28 January 2017: the Cochrane Central Register of Controlled Trials (CENTRAL), Embase, and PubMed. The search strategies are presented in Supplementary Table 1. We searched the following trial registers for ongoing trials: ClinicalTrials.gov (https://clinicaltrials.gov/), World Health Organization International Clinical Trials Registry Platform (http://apps.who.int/trialsearch/), Chinese Clinical Trial Registry (http://www.chictr.org.cn/searchproj.aspx). We also searched the following sources for conference reports and abstracts by handsearching and electronic searching from 2006 to 2016: Annual Meeting of the American Society of Clinical Oncology, Biennial Meeting of the International Journal of Gynecological Cancer Society, Biennial Meeting of the European Society of Gynaecological Oncology, and Annual Meeting on Women's Cancer of the Society of Gynecologic Oncology. In addition, we screened the reference lists from selected articles and relevant reviews retrieved in the initial search for additional studies.

### Eligibility criteria

We downloaded all titles and abstracts retrieved by electronic searching into the reference management database NoteExpress. Two reviewers (JY and QZ) removed duplicates and screened the remaining references independently. The full text of potentially relevant references were obtained and evaluated in detail to determine their eligibility. Any disagreements were resolved by discussion between the two reviewers or in consultation with a third reviewers (WX). Studies considered in this meta-analysis were either RCTs or non-randomized studies that met the following inclusion criteria: (1) evaluated exposure to statins and endometrial cancer risk, (2) reported risk ratio (RR) and a 95% confidence interval (CI) or provided data for their calculation. Articles were excluded if they were: (1) editorials, letters, reviews, and case reports; (2) studies without appropriate data for determining an estimate of RR and 95% CI. In cases of duplicate publications from the same population, only the largest and the most informative studies were included.

Studies reporting different measures of RR, including relative risk, rate ratio, odds ratio, hazard ratio, and incidence rate ratio, were included in this meta-analysis. Actually, these effect measures yield similar estimates of RR because of the low prevalence of endometrial cancer.

### Data extraction and quality assessment

Two reviewers (JY and QL) extracted data independently. The following variables were collected from each study: publication data (ie, first author’s name, publication year, and study location), study design, participants’age, sample size, RR and 95% CI, definition of statin exposure, and adjustment factors, if applicable. In non-randomized studies, when multiple estimates of effect (RR) were presented, the most adjusted estimate was extracted; when adjusted estimate was not available, crude estimate was extracted. As the important information was presented in all included studies, we did not contact the original authors for extra data or impute data for any outcomes.

Three reviewers (JY, QZ and QL) assessed the quality of included studies independently. The risk of bias in included RCTs was assessed using the Cochrane Collaboration’s tool [46]. It is a tool addressing seven domains: random sequence generation, allocation concealment, blinding of participants and personnel, blinding of outcome assessment, incomplete outcome data, selective reporting, and other issues. The quality of non-randomized studies was assessed according to the Newcastle–Ottawa scale (NOS), which uses a star system that ranges from 0 to 9 stars that judged on three aspects: the selection of the groups; the comparability of the groups; and the assessment of either the exposure or outcome of interest [47].

### Statistical analysis

For examining consistency of results across different study designs with different potential biases, we conducted two separate meta-analyses base on the study design: one meta-analysis of RCTs and a second meta-analysis of non-randomized studies. Then, we conducted a meta-analysis of all included studies.

Heterogeneity was measured using the Chi-square (χ^2^, or Chi^2^) and *I*^*2*^ test. When significant heterogeneity (*p* value < 0.10 or *I*^*2*^ > 50 %) was found, a random-effects model was applied to calculate the pooled effect; otherwise, a fixed-effects model was used. Once significant heterogeneity was found, we attempted to determine the causes of heterogeneity by examining individual study and conducting subgroup analysis by study design and study location if possible. To assess the stability of the results, we performed the leave-one-out sensitivity analysis. All analysis was performed using Stata version 12.0 software (Stata Corporation, College Station, TX).

## SUPPLEMENTARY MATERIALS TABLES



## References

[1] Siegel RL, Miller KD, Jemal A (2016). Cancer statistics, 2016. CA Cancer J Clin.

[2] Schachter M (2005). Chemical, pharmacokinetic and pharmacodynamic properties of statins: an update. Fundam Clin Pharmacol.

[3] Newman TB, Hulley SB (1996). Carcinogenicity of lipid-lowering drugs. JAMA.

[4] Keyomarsi K, Sandoval L, Band V, Pardee AB (1991). Synchronization of tumor and normal cells from G1 to multiple cell cycles by lovastatin. Cancer Res.

[5] Wong WW, Dimitroulakos J, Minden MD, Penn LZ (2002). HMG-CoA reductase inhibitors and the malignant cell: the statin family of drugs as triggers of tumor-specific apoptosis. Leukemia.

[6] Park HJ, Kong D, Iruela-Arispe L, Begley U, Tang D, Galper JB (2002). 3-hydroxy-3-methylglutaryl coenzyme A reductase inhibitors interfere with angiogenesis by inhibiting the geranylgeranylation of RhoA. Circ Res.

[7] Kusama T, Mukai M, Iwasaki T, Tatsuta M, Matsumoto Y, Akedo H, Inoue M, Nakamura H (2002). 3-hydroxy-3-methylglutaryl-coenzyme a reductase inhibitors reduce human pancreatic cancer cell invasion and metastasis. Gastroenterology.

[8] Schointuch MN, Gilliam TP, Stine JE, Han X, Zhou C, Gehrig PA, Kim K, Bae-Jump VL (2014). Simvastatin, an HMG-CoA reductase inhibitor, exhibits anti-metastatic and anti-tumorigenic effects in endometrial cancer. Gynecol Oncol.

[9] Kato S, Smalley S, Sadarangani A, Chen-Lin K, Oliva B, Brañes J, Carvajal J, Gejman R, Owen GI, Cuello M (2010). Lipophilic but not hydrophilic statins selectively induce cell death in gynaecological cancers expressing high levels of HMGCoA reductase. J Cell Mol Med.

[10] Ye X, Mneina A, Johnston JB, Mahmud SM (2017). Associations between statin use and non-Hodgkin lymphoma (NHL) risk and survival: a meta-analysis. Hematol Oncol.

[11] Singh S, Singh AG, Singh PP, Murad MH, Iyer PG (2013). Statins are associated with reduced risk of esophageal cancer, particularly in patients with Barrett's esophagus: a systematic review and meta-analysis. Clin Gastroenterol Hepatol.

[12] Singh S, Singh PP, Singh AG, Murad MH, Sanchez W (2013). Statins are associated with a reduced risk of hepatocellular cancer: a systematic review and meta-analysis. Gastroenterology.

[13] Singh PP, Singh S (2013). Statins are associated with reduced risk of gastric cancer: a systematic review and meta-analysis. Ann Oncol.

[14] Bonovas S, Filioussi K, Tsavaris N, Sitaras NM (2005). Use of statins and breast cancer: a meta-analysis of seven randomized clinical trials and nine observational studies. J Clin Oncol.

[15] Bonovas S, Filioussi K, Flordellis CS, Sitaras NM (2007). Statins and the risk of colorectal cancer: a meta-analysis of 18 studies involving more than 1.5 million patients. J Clin Oncol.

[16] Bonovas S, Filioussi K, Sitaras NM (2008). Statins are not associated with a reduced risk of pancreatic cancer at the population level, when taken at low doses for managing hypercholesterolemia: evidence from a meta-analysis of 12 studies. Am J Gastroenterol.

[17] Bonovas S, Nikolopoulos G, Filioussi K, Peponi E, Bagos P, Sitaras NM (2010). Can statin therapy reduce the risk of melanoma? A meta-analysis of randomized controlled trials. Eur J Epidemiol.

[18] Tan P, Zhang C, Wei SY, Tang Z, Gao L, Yang L, Wei Q (2016). Effect of statins type on incident prostate cancer risk: a meta-analysis and systematic review. Asian J Androl.

[19] Blais L, Desgagné A, LeLorier J (2000). 3-Hydroxy-3-methylglutaryl coenzyme A reductase inhibitors and the risk of cancer: a nested case-control study. Arch Intern Med.

[20] Clearfield M, Downs JR, Weis S, Whitney EJ, Kruyer W, Shapiro DR, Stein EA, Langendorfer A, Beere PA, Gotto AM (2001). Air Force/Texas Coronary Atherosclerosis Prevention Study (AFCAPS/TexCAPS): efficacy and tolerability of long-term treatment with lovastatin in women. J Womens Health Gend Based Med.

[21] Kaye JA, Jick H (2004). Statin use and cancer risk in the General Practice Research Database. Br J Cancer.

[22] Strandberg TE, Pyörälä K, Cook TJ, Wilhelmsen L, Faergeman O, Thorgeirsson G, Pedersen TR, Kjekshus J, 4S Group (2004). Mortality and incidence of cancer during 10-year follow-up of the Scandinavian Simvastatin Survival Study (4S). Lancet.

[23] Friedman GD, Flick ED, Udaltsova N, Chan J, Quesenberry CP, Habel LA (2008). Screening statins for possible carcinogenic risk: up to 9 years of follow-up of 361,859 recipients. Pharmacoepidemiol Drug Saf.

[24] Haukka J, Sankila R, Klaukka T, Lonnqvist J, Niskanen L, Tanskanen A, Wahlbeck K, Tiihonen J (2010). Incidence of cancer and statin usage–record linkage study. Int J Cancer.

[25] Leung HW, Chan AL, Lo D, Leung JH, Chen HL (2013). Common cancer risk and statins: a population-based case-control study in a Chinese population. Expert Opin Drug Saf.

[26] Coogan PF, Rosenberg L, Strom BL (2007). Statin use and the risk of 10 cancers. Epidemiology.

[27] Yu O, Boudreau DM, Buist DS, Miglioretti DL (2009). Statin use and female reproductive organ cancer risk in a large population-based setting. Cancer Causes Control.

[28] Fortuny J, Sima C, Bayuga S, Wilcox H, Pulick K, Faulkner S, Zauber AG, Olson SH (2009). Risk of endometrial cancer in relation to medical conditions and medication use. Cancer Epidemiol Biomarkers Prev.

[29] Jacobs EJ, Newton CC, Thun MJ, Gapstur SM (2011). Long-term use of cholesterol-lowering drugs and cancer incidence in a large United States cohort. Cancer Res.

[30] Lavie O, Pinchev M, Rennert HS, Segev Y, Rennert G (2013). The effect of statins on risk and survival of gynecological malignancies. Gynecol Oncol.

[31] Sperling CD, Verdoodt F, Friis S, Dehlendorff C, Kjaer SK (2017). Statin use and risk of endometrial cancer: a nationwide registry-based case-control study. Acta Obstet Gynecol Scand.

[32] Boudreau DM, Yu O, Johnson J (2010). Statin use and cancer risk: a comprehensive review. Expert Opin Drug Saf.

[33] Gonyeau MJ (2014). The spectrum of statin therapy in cancer patients: is there a need for further investigation?. Curr Atheroscler Rep.

[34] Graaf MR, Beiderbeck AB, Egberts AC, Richel DJ, Guchelaar HJ (2004). The risk of cancer in users of statins. J Clin Oncol.

[35] Friis S, Poulsen AH, Johnsen SP, McLaughlin JK, Fryzek JP, Dalton SO, Sørensen HT, Olsen JH (2005). Cancer risk among statin users: a population-based cohort study. Int J Cancer.

[36] Karp I, Behlouli H, Lelorier J, Pilote L (2008). Statins and cancer risk. Am J Med.

[37] Farwell WR, Scranton RE, Lawler EV, Lew RA, Brophy MT, Fiore LD, Gaziano JM (2008). The association between statins and cancer incidence in a veterans population. J Natl Cancer Inst.

[38] Vinogradova Y, Coupland C, Hippisley-Cox J (2011). Exposure to statins and risk of common cancers: a series of nested case-control studies. BMC Cancer.

[39] Bonovas S, Filioussi K, Tsavaris N, Sitaras NM (2006). Statins and cancer risk: a literature-based meta-analysis and meta-regression analysis of 35 randomized controlled trials. J Clin Oncol.

[40] Dale KM, Coleman CI, Henyan NN, Kluger J, White CM (2006). Statins and cancer risk: a meta-analysis. JAMA.

[41] Kuoppala J, Lamminpää A, Pukkala E (2008). Statins and cancer: a systematic review and meta-analysis. Eur J Cancer.

[42] Verdoodt F, Friis S, Dehlendorff C, Albieri V, Kjaer SK (2016). Non-steroidal anti-inflammatory drug use and risk of endometrial cancer: a systematic review and meta-analysis of observational studies. Gynecol Oncol.

[43] Zhang D, Bai B, Xi Y, Zhao Y (2016). Can aspirin reduce the risk of endometrial cancer?: a systematic review and meta-analysis of observational studies. Int J Gynecol Cancer.

[44] Kunz R, Oxman AD (1998). The unpredictability paradox: review of empirical comparisons of randomised and non-randomised clinical trials. BMJ.

[45] Moher D, Liberati A, Tetzlaff J, Altman DG, PRISMA Group (2010). Preferred reporting items for systematic reviews and meta-analyses: the PRISMA statement. Int J Surg.

[46] Higgins JP, Altman DG, Gøtzsche PC, Jüni P, Moher D, Oxman AD, Savovic J, Schulz KF, Weeks L, Sterne JA, Cochrane Bias Methods Group, Cochrane Statistical Methods Group (2011). The Cochrane Collaboration's tool for assessing risk of bias in randomised trials. BMJ.

[47] Wells GA, Shea B, O’Connell D, Peterson J, Welch V, Losos M, Tugwell P http://www.ohri.ca/programs/clinical_epidemiology/oxford.asp.

