# Supplementary material for: Statin use and endometrial cancer risk: a meta-analysis
Source: Oncotarget. 2017 Jun 27;8(37):62425–34. doi: 10.18632/oncotarget.18658 (PMC5617516; doi:10.18632/oncotarget.18658)
Supplement: Supplementary file 1 [file oncotarget-08-62425-s001.pdf]

Statin use and endometrial cancer risk: a meta-analysis

SUPPLEMENTARY MATERIALS

Supplementary Table 1: Search strategy

**Pubmed search strategy**	
#1	"Hydroxymethylglutaryl-CoA Reductase Inhibitors"[Pharmacological Action
#2	"Hydroxymethylglutaryl-CoA Reductase Inhibitors"[Mesh]
#3	hydroxymethylglutaryl-coa reductase inhibitor*
#4	HMG-CoA reductase inhibitor* OR statin*
#5	atorvastatin* OR cerivastatin* OR fluvastatin* OR lovastatin* OR mevastatin* OR pitavastatin* OR pravastatin* OR rivastatin* OR rosuvastatin* OR simvastatin*
#6	#1 OR #2 OR #3 OR #4 OR #5
#7	"Endometrial Neoplasms"[Mesh]
#8	endometr*
#9	neoplas* OR carcinom* OR malignan* OR cancer* OR tumor* OR tumour*
#10	#8 AND #9
#11	#7 OR #10
#12	#6 AND #11
**CENTRAL search strategy**	
#1	MeSH descriptor: [Hydroxymethylglutaryl-CoA Reductase Inhibitors] explode all trees
#2	"hydroxymethylglutaryl-CoA reductase inhibitor*" OR "HMG-CoA reductase inhibitor*"
#3	"HMG CoA*" OR "HMG-CoA*" OR statin*
#4	atorvastatin* OR cerivastatin* OR fluvastatin* OR lovastatin* OR mevastatin* OR pitavastatin* OR pravastatin* OR rivastatin* OR rosuvastatin* OR simvastatin*
#5	#1 OR #2 OR #3 OR #4
#6	MeSH descriptor: [Endometrial Neoplasms] explode all trees
#7	endometr* near/5 neoplas* in Trials
#8	endometr* near/5 malignan* in Trials
#9	endometr* near/5 cancer* in Trials
#10	endometr* near/5 tumor* in Trials
#11	endometr* near/5 tumour* in Trials
#12	#6 OR #7 OR #8 OR #9 OR #10 OR #11
#13	#5 AND #12
**Embase search strategy**	
#1	'hydroxymethylglutaryl coenzyme a reductase inhibitor'/exp
#2	'hydroxymethylglutaryl coenzyme a reductase inhibitor*' OR 'hmg-coa reductase inhibitor*' OR statin*
#3	atorvastatin* OR cerivastatin* OR fluvastatin* OR lovastatin* OR mevastatin* OR pitavastatin* OR pravastatin* OR rivastatin* OR rosuvastatin* OR simvastatin*
#4	#1 OR #2 OR #3
#5	'endometrium tumor'/exp
#6	endometr*
#7	neoplas* OR carcinom* OR malignan* OR cancer* OR tumor* OR tumour*
#8	#6 AND #7
#9	#5 OR #8
#10	#4 AND #9
